# CANreduce 2.0 Adherence-Focused Guidance for Internet Self-Help Among Cannabis Users: Three-Arm Randomized Controlled Trial

**DOI:** 10.2196/27463

**Published:** 2021-04-30

**Authors:** Christian Baumgartner, Michael Patrick Schaub, Andreas Wenger, Doris Malischnig, Mareike Augsburger, Marc Walter, Thomas Berger, Lars Stark, David Daniel Ebert, Matthew T Keough, Severin Haug

**Affiliations:** 1 Swiss Research Institute for Public Health and Addiciton University of Zurich Zürich Switzerland; 2 Institute for Addiction Prevention Addiction and Drug Coordination Vienna Vienna Austria; 3 University Psychiatric Clinics University of Basel Basel Switzerland; 4 Department of Clinical Psychology and Psychotherapy University of Bern Bern Switzerland; 5 Arud Centre for Addiction Medicine Zurich Switzerland; 6 Department for Sport and Health Sciences, Chair for Psychology and Digital Mental Health Care Technical University Munich Munich Germany; 7 Department of Psychology York University Toronto, ON Canada

**Keywords:** cannabis, common mental disorders, adherence, social presence, internet, cognitive behavioral therapy, motivational interviewing, therapy, mental health, mental disorder, adherence, guidance, self-help, drug abuse, randomized controlled trial

## Abstract

**Background:**

Despite increasing demand for treatment among cannabis users in many countries, most users are not in treatment. Internet-based self-help offers an alternative for those hesitant to seek face-to-face therapy, though low effectiveness and adherence issues often arise.

**Objective:**

Through adherence-focused guidance enhancement, we aimed to increase adherence to and the effectiveness of internet-based self-help among cannabis users.

**Methods:**

From July 2016 to May 2019, cannabis users (n=775; male: 406/575, 70.6%, female: 169/575, 29.4%; age: mean 28.3 years) not in treatment were recruited from the general population and were randomly assigned to (1) an adherence-focused guidance enhancement internet-based self-help intervention with social presence, (2) a similar intervention with an impersonal service team, and (3) access to internet as usual. Controls who were placed on a waiting list for the full intervention after 3 months underwent an assessment and had access to internet as usual. The primary outcome measurement was cannabis-use days over the preceding 30 days. Secondary outcomes included cannabis-dependence severity, changes in common mental disorder symptoms, and intervention adherence. Differences between the study arms in primary and secondary continuous outcome variables at baseline, posttreatment, and follow-up were tested using pooled linear models.

**Results:**

All groups exhibited reduced cannabis-use days after 3 months (social presence: –8.2 days; service team: –9.8 days; internet as usual: –4.2 days). The participants in the service team group (*P*=.01, *d*=.60) reported significantly fewer cannabis-use days than those in the internet as usual group; the reduction of cannabis use in the social presence group was not significant (*P*=.07, *d*=.40). There was no significant difference between the 2 intervention groups regarding cannabis-use reduction. The service team group also exhibited superior improvements in cannabis-use disorder, cannabis-dependence severity, and general anxiety symptoms after 3 months to those in the internet as usual group.

**Conclusions:**

The adherence-focused guidance enhancement internet-based self-help intervention with an impersonal service team significantly reduced cannabis use, cannabis-use disorder, dependence severity, and general anxiety symptoms.

**Trial Registration:**

ISRCTN Registry ISRCTN11086185; http://www.isrctn.com/ISRCTN11086185

## Introduction

Cannabis is the most consumed illicit drug in Europe, having witnessed a steady increase in recent years, evidenced by roughly 24.7 million European users in 2019 [1]. The global number of cannabis users was estimated as 178 million people in 2017 [2]. As more and more countries consider decriminalization or outright legalization, it seems unlikely that the increase in cannabis users will stagnate soon [3]. However, only a minority of cannabis users seem to develop cannabis dependence; in general population surveys, the risk of becoming dependent on cannabis appears to be between 10% and 11% of all cannabis users [4,5]. However, for cannabis users who start at a young age, the risks of cannabis dependence [6] and cannabis use problems [7] are significantly higher. In addition, poorer mental and physical health, lower educational attainment, and reduced cognitive performance than non-cannabis users are common among daily cannabis users [8]. Numerous studies [9] also point to a broad range of often co-occurring mental health disorders, such as depression, anxiety, and posttraumatic stress disorder, during the treatment of problematic cannabis use.

Treatment demand in Europe for first-time admissions with cannabis listed as the main problem substance has been increasing steadily, having almost doubled from roughly 45,000 in 2006 to approximately 83,000 in 2017 [1]. However, it is clear that, although the number of clients seeking treatment has increased, they still account for just a small minority of cannabis users who could potentially benefit from treatment, with or without comorbid mental health problems [9]. Similarly, only a few consumers seek professional medical assistance [10], suggesting that a broader range of treatment options should be provided [11]. Various potential barriers prevent people from seeking treatment, including poor accessibility to treatment centers, the lack of awareness of negative health consequences, the wish to reduce cannabis use on their own [12], and fear of stigmatization as a drug addict, which seems to be a major factor [13,14]. Facilitators of treatment, on the other hand, include improving available information, increased access to cannabis-specific services, providing additional treatment options, and making admissions easier [15], all of which many internet-based interventions could provide.

Studies on web-based interventions for which participants were recruited from the general adult population (>18 years old) have been shown to draw a cannabis-using population that is different than those entering outpatient addiction treatment centers, not only in terms of having a higher level of education and being older, but also in terms of reporting more frequent cannabis use [14,16]. However, poor adherence to the intervention is often found in these studies [17,18]. Moreover, a recent meta-analysis [19] on internet-based treatments for cannabis users yielded significant but only small effect sizes for the reduction of cannabis use (mostly frequency) in the short term (15 comparisons, Hedges g=0.12) that could not be maintained longer term (12 months). The effects of multisession interventions, such as those combining cognitive behavioral therapy [20] with motivational interviewing [21], produced larger effect sizes (6 comparisons, Hedges g=0.18) than single-session interventions using approaches like brief interventions [22] and motivational interviewing (13 comparisons, Hedges g=0.09). Among the studies assessing multisession interventions, only 2 took symptoms of possible co-occurring mental health disorders into account [14,23].

In previous studies [13,14], called CANreduce 1.0, we were able to show that additional professional chat sessions increased the effectiveness of an internet-based self-help program designed to reduce cannabis use. The study [14] also found that participants who had the opportunity but did not participate in these chat sessions, nevertheless reduced their cannabis use more than those who only received internet-based self-help from the beginning. It seems that, on its own, having a professional therapist send chat invitations helped to reduce cannabis use in cannabis users. Since only a quarter of the participants in the treatment arm with chat took part in at least one chat appointment, we wondered whether the same effect could be achieved by replacing the professional therapist with a virtual eCoach. We also found that almost half (44.8%) of participants screened positive for clinically relevant depression symptoms at baseline [14]. Comorbidity of depressive symptoms and substance use and its hindrance on positive treatment outcomes has repeatedly been demonstrated [24].

CANreduce 2.0, a minimally guided internet-based self-help intervention for cannabis users, is designed to overcome the issues of low intervention adherence and effectiveness, as well as to address frequently co-occurring mental health disorders. This intervention is based on adherence-focused guidance which has, to date, never been tested as a component of an internet intervention for individuals with a substance use disorder but has been documented to be effective at increasing adherence to web-based self-help for the reduction of stress and depression symptoms [25,26]. The concept of adherence-focused guidance enhancement is primarily based on the supportive-accountability model of guidance in web-based interventions [27], which argues that adherence to internet-based interventions relies on an online coach (eCoach) who is seen as trustworthy, benevolent, and having expertise, and who has clear, process-oriented expectations in a reciprocal eCoach–participant relationship. In addition to an eCoach, we incorporated cognitive behavioral therapy–based approaches [28-31] into the program to target issues that potentially help to ameliorate overlapping common mental disorder symptoms, such as inactivity, depressed mood, excessive rumination, and difficulty relaxing.

The primary goal of this study was to investigate whether intervention effectiveness and program adherence can be increased by implementing adherence-focused guidance and emphasizing the social presence factor of a personal eCoach when compared with a general support team implementation.

## Methods

### Study Design

This study was a 3-arm, randomized controlled trial that compared 2 versions of a minimally guided web-based self-help intervention for cannabis users based on adherence-focused guidance, cognitive behavioral therapy, motivational interviewing, and social presence factor—one combined with a personal eCoach (social presence), and one with a general support team (service team)—to a wait-list control group that underwent an assessment and had access to internet as usual (internet as usual) for the purpose of reducing cannabis use and associated mental health problems. The internet as usual group was able to use the internet to search for additional support and information regarding cannabis use from other online resources. Each intervention lasted for 6 weeks and was followed by a posttreatment survey and a follow-up survey 3 months postbaseline.

Participants were randomized, by computer, to the 3 conditions in a 1:1:1 ratio. Participants in the social presence and service team groups did not know to which program version they had been assigned, while participants in the internet as usual group knew they had been assigned to treatment as usual. The study was approved by the ethics committee of the Canton of Zurich on July 4, 2016 (BASEC 2016-00264) and registered (ISRCTN11086185). A detailed study protocol has been published [32].

### Recruitment and Inclusion and Exclusion Criteria

We recruited participants from August 2016 through May 2019 with 2 websites, advertisements in relevant internet forums and newspapers (or online versions thereof), and search engine website advertisements. Study inclusion and exclusion criteria, and the rationale behind them, are summarized in Table 1.

**Table 1:** Inclusion and exclusion criteria and underlying rationale.

Criteria			Reasoning
**Inclusion**			
	Informed consent via the web form	To ensure knowledge of procedures and the declaration of consent	
	Minimum age: 18 years	To ensure a minimum age of participation	
	Cannabis use at least once weekly over the last 30 days	To include participants with less than daily cannabis use, increase validity	
	At least once weekly internet access and a valid email address	To ensure at least some access to the intervention	
	Good command of the German language	To ensure that participants will be able to understand the information provided	
**Exclusion**			
	Participation in other psychosocial or pharmacological treatments for the reduction or cessation of cannabis use	To avoid confounding treatment effects	
	Current pharmacologically treated psychiatric disease or any history of psychosis, schizophrenia, bipolar type I disorder or significant current suicidal or homicidal thoughts	To avoid having participants with these problems enter the study	

For compensation, all participants who completed the final follow-up evaluation were offered a choice of either an online voucher worth 30 € (approximately US $35.85) or donating that amount to charity.

### Sample Size Calculation

We anticipated that a Cohen *d*=0.30 was appropriate, by employing previous internet-based studies in cannabis users [16,32] recruited from the general population, and adherence-focused guidance enhancement internet-based studies among individuals with stress or depression [25,26] to estimate effect-size differences between the adherence-focused guidance-enhanced version with (social presence) versus without (service team) a personal eCoach. This resulted in a sample size of 176 for each study arm (n=528 in total) to detect a small effect size with 80% power and an alpha error of 5% (2-tailed testing).

### Treatment Arms

Both active interventions—social presence and service team—consisted of a dashboard and 8 self-help intervention modules that included stories of 6 fictional companions who appeared within the modules at key points, with the goal of encouraging reflection on potential questions raised by the modules. Table 2 provides an overview of the modules’ contents and underlying therapeutic approaches. Both active interventions also incorporated a use and activity diary, weekly semiautomated motivational and adherence-focused guidance–based email feedback, and a section containing educational information on cannabis and health. The semiautomated motivational emails were triggered by a moderator, depending on how participants responded in exercises. These feedback emails also included module suggestions, dealing with high-risk situations, cravings, or the pros and cons of their use. Participants in both active intervention groups also were invited to ask questions of either their eCoach (social presence group) or support team (service team group) whenever they felt the need.

**Table 2:** Modules.

Module	Content	Therapeutic approach
Module 1: Introduction	General overview Introduction of fictional companions Reflection on personal cannabis use	Based on motivational interviewing techniques [21]
Module 2 : Identifying risk situations	Identifying personal high-risk situations Recognizing seemingly irrelevant, but triggering decisions	Cognitive behavioral therapy approach to relapse Prevention [28]
Module 3: Working on needs	Strengthening social contacts Decreasing excessive ruminations Developing healthier sleeping habits	Behavioral activation approach [29]
Module 4: Craving	Concept of craving Ways to deal with feelings of craving	Based on cognitive behavioral therapy [30]
Module 5: Dealing with relapses	Relapse prevention Dealing with relapses	Cognitive behavioral therapy approach to Relapse Prevention [28]
Module 6 : Working on problems	Relationships between use, problems, and depressive symptoms Skills to deal with solvable and unsolvable problems	Social problem-solving approach [31]
Module 7: Saying “no”; refusal skills	Strengthening refusal skills for use in high-risk situations	Based on cognitive behavioral therapy [30]
Module 8: Preserving achievements	Review of program List of 5 personalized points to help secure achievements after the program is complete	Based on motivational interviewing techniques [21]

The only difference between the social presence and service team interventions was that, for the social presence group, a semiautomated eCoach, with short personal introduction videos (Multimedia Appendix 1) that preceded most of the modules and a picture of the female eCoach displayed on the dashboard, was used, while for the service team group, an anonymous semiautomated support team, with no pictures, videos, or any other kind of social presence, was used.

The control group had access to the internet as usual, since it was deemed impossible and unethical to prevent participants in this group from seeking out other internet support or face-to-face treatment options during the waiting period. A detailed description of the groups and their technical specifications is provided in the study protocol [32].

CANreduce 2.0 is regarded as a medical device and is CE (*Conformité Européenne*) certified.

### Measurements

Table 3 provides an overview of the measurement instruments. The primary outcome of interest was the number of days of cannabis use over the preceding 30 days, in accordance with the timeline follow-back method [33,34]. Secondary outcomes included the severity of cannabis-use disorder assessed using the Cannabis Use Disorders Identification Test-Revised (CUDIT-R [35]); the Severity of Dependence Scale (SDS [36]); quantity of cannabis use over the previous 30 days using the timeline follow-back method, quantified in individually standardized cannabis joint sizes (detailed description in the study protocol [32]); the use of alcohol, tobacco, or other illicit drugs besides cannabis (with questions derived from the European Adaptation of a Multidimensional Assessment Instrument for Drug and Alcohol Dependence [37]); change in depression (Centre of Epidemiologic Studies of Depression, CES-D, scale [38], anxiety (Generalized Anxiety Disorder–7, GAD-7 [39]), and attention deficit and hyperactivity symptoms (adult Attention Deficit and Hyperactivity Self-Report Scale, ASRS, version 1.1) [40]; Short Screening Scale for lifetime Diagnostic and Statistical Manual of Mental Disorders, fourth edition, Posttraumatic Stress Disorder [41]; client satisfaction questionnaire [42]; Working Alliance Inventory adapted for web-based interventions [43], and treatment adherence (finished modules, time spent on modules). Furthermore, the occurrence of any negative intervention effects was identified using a questionnaire [44] at the 3-month follow-up assessment. We asked all participants if they had used any treatment other than CANreduce during the 3 months and, if so, to identify it from a predefined list of services. Details regarding study measures are reported in the study protocol [32].

**Table 3:** Assessment instruments.

Assessment instruments	Assessment
Sociodemographic data	Baseline
Center for Epidemiologic Studies Depression scale (range 0-60)	Baseline
Short Screening Scale for DSM-IV^a^ Posttraumatic Stress Disorder (range 7-28)	Baseline
General Anxiety Disorder–7 (range 0-21)	Baseline
Adult ADHD^b^ Self-Report Scale version 1.1 (range 0-24)	Baseline
Frequency of cannabis use (last 30 days)	Baseline, posttreatment, 3-months follow-up
Cannabis use according to the timeline follow-back method	Baseline, posttreatment, 3-months follow-up
Cannabis Use Disorder Identification Test-Revised (range 0-40)	Baseline, posttreatment, 3-months follow-up
Severity of Dependence Scale (range 0-15)	Baseline, posttreatment, 3-months follow-up
*Fragebogen Substanzanamnese* (substance use questionnaire)	Baseline, posttreatment, 3-months follow-up
Client Satisfaction Questionnaire-I (range 8-32)	Posttreatment
Intervention adherence (continuous assessment over 6-week treatment)	Posttreatment
WAI-TECH^c^ (range 10-70)	Posttreatment
Negative effects [44]	3 months follow-up

^a^DSM-IV: Diagnostic and Statistical Manual of Mental Disorders, fourth edition.

^b^ADHD: attention deficit hyperactivity disorder.

^c^Working Alliance Inventory adapted for web-based interventions.

### Statistical Analysis

Data were analyzed according to intention-to-treat (ITT). To address missing data for the ITT analyses, we applied multiple imputation procedures using the multivariate imputation by chained equations software package [45] in R (version 3.6.1; R Foundation for Statistical Computing), a minor deviation from the study protocol, which involves specifying a multivariate distribution for the missing data and drawing imputations from their conditional distributions using Markov chain Monte Carlo techniques. As recommended, 20 imputation sets were employed [45]. All sociodemographic, as well as primary and secondary outcome variables that had been assessed in all 3 groups, were included in the imputation. Reported outcomes use the ITT results from the imputed data sets, but complete case analysis results are also reported.

Cohen *d* effect size was used to compare change scores between the 3 treatment arms. As suggested elsewhere, Cohen *d*=0.2 indicates a small effect, Cohen *d*=0.5 indicates a medium effect, and Cohen *d*=0.8 indicates a large effect [46].

Differences between the study arms in primary and secondary continuous outcome variables at baseline, posttreatment, and follow-up were tested using pooled linear models. Change scores from baseline for primary and secondary outcomes were dependent variables, and study condition was the independent variable; all controlled for the baseline value of the respective outcome variable. Effect sizes were calculated for changes from baseline to follow-up (within-group effect size *d*_w_) and between the 2 intervention groups (social presence, service team) and internet as usual. All *P* values are 2-sided with no adjustment made for multiple comparisons, which was deemed unnecessary based on CONSORT recommendations [47].

## Results

### Participation

**Figure 1:**
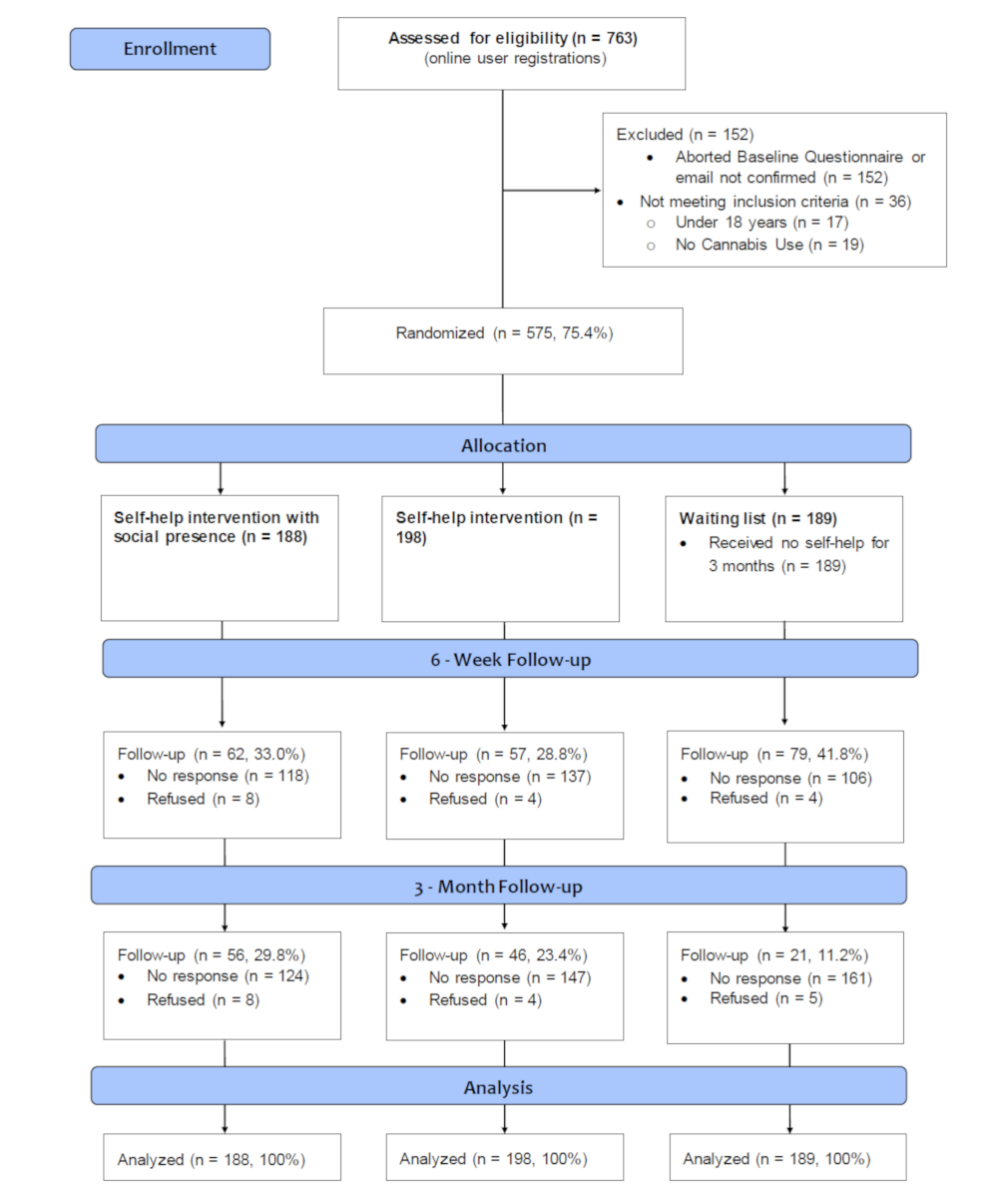
Participation flowchart.

Between July 2016 and May 2019, a total of 763 people registered online for the program, among whom 575 were randomized to the 3 study arms (Figure 1). All participants received email reminders for follow-ups and subsequent telephone calls if they did not complete the survey. We were able to reach 198 participants (34.4% of the initial sample) posttreatment, and this number dropped to 123 (21.4%) for the final assessment 3 months postbaseline. A coding bug arose within the email system which affected distribution of the final assessment questionnaire to the internet as usual group (only telephone follow-ups were performed). There were significant differences in follow-up rate between the study groups (χ^2^=20.16, *P<*.001) that may have been caused by this bug.

### Baseline Characteristics

Of the 575 participants, 406 (70.6%) were male, and the average age was 28.3 (SD 7.9). Most (n=234, 40.7%) were from Switzerland, followed closely by 216 (37.6%) from Austria, and 121 (21.0%) from Germany. The average participant had used cannabis almost daily (25.7 days, SD 5.9) over the preceding 30 days. Complete case analyses of baseline data and study group comparisons are summarized in Table 4.

**Table 4:** Baseline participant data.

Characteristic			Social presence (n=188)		Service team (n=198)		Internet as usual (n=189)		All (N=575)		*F* test (*df1*,*df2*)		*P* value
**Gender, n (%)**											0.57 (2,575)^a^		.75
	Female	52 (27.7)		58 (29.3)		59 (31.2)		169 (29.4)					
	Male	136 (72.3)		140 (70.7)		130 (68.8)		406 (70.6)					
Age, mean (SD)			28.0 (7.4)		27.9 (7.9)		28.9 (8.3)		28.3 (7.9)		1.01 (2,572)		.36
**Highest education, n (%)**											14.20 (10,575)^a^		.16
	Primary school	12 (6.4)		17 (8.6)		10 (5.3)		39 (6.8)					
	Apprenticeship	31 (16.5)		36 (18.2)		53 (28.0)		120 (20.9)					
	Secondary school	64 (34.0)		56 (28.3)		46 (24.3)		166 (28.9)					
	Technical college	25 (13.3)		32 (16.2)		27 (14.3)		84 (14.6)					
	University	49 (26.1)		48 (24.2)		49 (25.9)		146 (25.4)					
	Not specified	7 (3.7)		9 (4.5)		4 (2.1)		20 (3.5)					
**Country of origin, n (%)**											9.73 (6,575)^a^		.14
	Switzerland	74 (39.4)		77 (38.9)		83 (43.9)		234 (40.7)					
	Austria	67 (35.6)		72 (36.4)		77 (40.7)		216 (37.6)					
	Germany	45 (23.9)		49 (24.7)		27 (14.3)		121 (21.0)					
	Other	2 (1.1)		0 (0.0)		2 (1.1)		4 (0.7)					
Centre for Epidemiological Studies Depression scale, mean (SD)			20.4 (10.0)		23.5 (11.1)		21.7 (10.1)		21.9 (10.5)		4.48 (2,572)		.01
Generalized Anxiety Disorder–7, mean (SD)			7.4 (4.8)		7.9 (5.0)		7.6 (4.5)		7.7 (4.8)		0.55 (2,571)		.58
Cannabis-use disorder, mean (SD)			20.8 (5.5)		20.7 (5.8)		21.6 (5.3)		21.0 (5.5)		1.41 (2,572)		.24
Severity of Dependence scale, mean (SD)			7.4 (3.1)		8.1 (3.3)		8.1 (3.2)		7.9 (3.2)		3.16 (2,572)		.04
Adult ADHD^b^ Self-Report Scale, mean (SD)			10.7 (3.9)		10.8 (4.1)		10.8 (4.2)		10.8 (4.1)		0.09 (2,572)		.92
Short Screening Scale for PTSD^c^, mean (SD)			13.2 (4.5)		12.8 (5.2)		13.8 (5.6)		13.2 (5.1)		0.56 (2,174)		.57
Number of cannabis joints, mean (SD)			22.6 (16.0)		21.3 (15.2)		23.6 (17.7)		22.5 (16.3)		0.89 (2,572)		.41
Number of cannabis-use days, mean (SD)			24.9 (6.7)		26.1 (5.3)		26.2 (5.5)		25.7 (5.9)		2.65 (2,572)		.07
**Number of years of use, mean (SD)**													
	Cannabis	8.5 (7.2)		7.6 (6.6)		9.1 (6.7)		8.4 (6.8)		2.62 (2,569)		.07	
	Alcohol	5.5 (7.0)		4.7 (6.2)		5.4 (7.1)		5.2 (6.8)		0.82 (2,525)		.44	
	Alcohol risky use^d^	1.7 (3.9)		1.6 (3.7)		1.9 (4.3)		1.7 (3.9)		0.19 (2,500)		.83	
	Cocaine	0.6 (2.6)		0.4 (2.2)		0.3 (1.0)		0.4 (2.0)		1.14 (2,496)		.32	

^a^Chi-square test (*df*).

^b^ADHD: attention deficit hyperactivity disorder.

^c^PTSD: posttraumatic stress disorder.

^d^Risky use is defined as 5 or more standard drinks per day on at least 3 days a week. A standard drink is defined as 50 mL spirits, 150-200 mL wine, or 330-450 mL beer.

### Primary Outcome: Cannabis-Use Days

**Figure 2:**
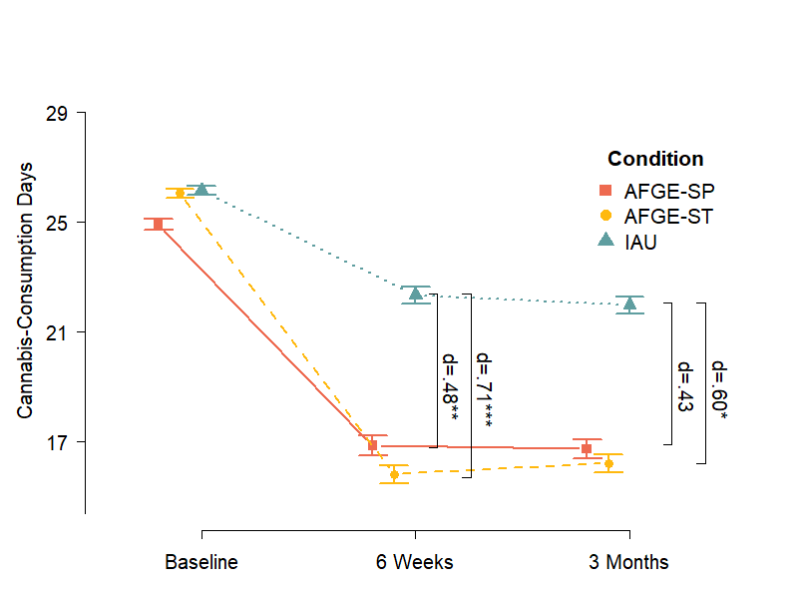
Cannabis use in the previous 30 days. AFGE-SP: adherence-focused guidance enhancement with social presence; AFGE-ST: adherence-focused guidance enhancement with service team; IAU: internet as usual.

Immediately posttreatment, both intervention groups (social presence: mean 8.0, SD 9.3, *d*_w_=.89; service team: mean 10.7 days, SD 9.5, *d*_w_=1.18) reduced their cannabis use significantly more than internet as usual (mean 3.8, SD 8.1, *d*_w_=.55) (social presence: B=–4.34, CI –7.21 to –1.47, *P*=.004, between-group effect size *d*=.48; service team: B=–6.43, CI –9.87 to –2.97, *P<*.001, *d*=.71). These effects persisted 3 months postbaseline, with participants in the service team (mean 9.8, SD 9.9, *d*_w_=1.18) group still reducing their cannabis-use days significantly more (B=–5.70, CI –10.09 to –1.30, *P*=.01, *d*=.60) than in the control group (mean 4.2 days, SD 8.8, *d*_w_=.55). Similarly, there was a significantly greater reduction in the social presence group (mean 8.2 days, SD 9.8, *d*_w_=.93) than in the control group (B=–4.41, CI –9.19 to 0.37, *P*=.07, *d*=.40). There was no significant difference between the 2 intervention groups immediately posttreatment (*P*=.26) or 3 months postbaseline (*P*=.44) (Figure 2; Table 5).

**Table 5:** Regression analysis (intention to treat).

Variable	Social presence			Service team	
	B (95% CI)	*P* value	B (95% CI)		*P* value
Consumption days^a^	–4.41 (–9.19, 0.37)	.07	–5.70 (–10.09, –1.32)		.01
Cannabis Use Disorder Identification Test	–2.15 (–4.49, 0.18)	.07	–3.39 (–5.96, –0.83)		.01
Severity of Dependence Scale	–1.49 (–3.45, 0.47)	.13	–2.16 (–3.90, –0.42)		.02
Generalized Anxiety Disorder–7	–2.07 (–3.59, –0.55)	.001	–2.87 (–4.43, –1.31)		<.001
Adult ADHD^b^ Self-Report Scale	0.63 (–1.68, 2.94)	.57	–0.51 (–2.59, 1.56)		.61
Centre for Epidemiological Studies Depression scale	0.41 (–3.49, 4.32)	.83	–1.76 (–5.99, 2.47)		.40

^a^Previous 30 days according to the timeline follow-back method.

^b^ADHD: attention deficit hyperactivity disorder.

### Secondary Outcomes

At follow-up 3 months postbaseline, a significant difference was noted in the decrease in cannabis-use disorder severity between the service team group and controls (B=–3.39, CI –5.96 to –0.83, *P*=.01, *d*=.52), but no significant difference was detected between the social presence and the internet as usual group (*P*=.07). Additionally, there was a significantly greater reduction in the severity of dependence in the service team group compared to the internet as usual group (B=–2.16, CI –3.90 to –0.42, *P*=.02, *d*=.60). There were significantly greater reductions in general anxiety disorder symptoms in both groups (social presence: B=–2.70, CI –3.59 to –0.55, *P*=.001, *d*=.41; service team: B=–2.87, CI –4.43 to –1.31, *P<*.001, *d*=.51) compared to the internet as usual group. All groups decreased their mean ASRS score, but no significant intergroup differences were detected (social presence: mean 0.9, SD 3.9, *P*=.57; service team: mean 2.1, SD 4.2, *P*=.61; internet as usual: mean 1.6, SD 4.0). Similarly, mean CES-D scores decreased in all groups (social presence: mean 3.6, SD 9.6, *P*=.83; service team: mean 7.5, SD 10.3, *P*=.40; internet as usual: mean 4.7, SD 10.4) with no significant between-group differences (Table 6 and Table 7). Upon review, the number of standard joints was deemed unreliable as a measurement and was dropped from analysis.

**Table 6:** Complete case analysis.

Group and measure			Baseline		Posttreatment				Follow-up		
		Mean (SD)		Mean (SD)		*d*^a^ (95% CL)		Mean (SD)		*d* (95% CL)	
**Internet as usual (n=189)**											
	Use days^b^	26.16 (5.53)		22.58 (9.29)		N/A^c^		21.81 (8.30)		N/A	
	CUDIT^d^	21.57 (5.30)		20.11 (6.49)		N/A		18.48 (5.61)		N/A	
	SDS^e^	8.12 (3.16)		7.79 (3.41)		N/A		7.00 (3.33)		N/A	
	GAD-7^f^	7.61 (4.54)		N/A		N/A		7.14 (2.53)		N/A	
	ASRS^g^	10.84 (4.21)		N/A		N/A		9.71 (4.22)		N/A	
	CES-D^h^	21.69 (10.12)		N/A		N/A		15.67 (6.19)		N/A	
**Social presence (n=188)**											
	Use days	24.92 (6.67)		15.74 (10.95)		0.53 (0.18, 0.85)		16.54 (10.51)		0.32 (–0.19, 0.71)	
	CUDIT	20.79 (5.46)		16.90 (6.14)		0.54 (0.19, 0.87)		14.88 (6.73)		0.61 (0.09, 1.11)	
	SDS	7.38 (3.10)		5.85 (3.26)		0.37 (0.03, 0.70)		5.14 (3.15)		0.39 (–0.12, 0.89)	
	GAD-7	7.42 (4.79)		N/A		N/A		5.26 (3.74)		0.19 (–0.32, 0.69)	
	ASRS	10.69 (3.89)		N/A		N/A		9.46 (3.66)		–0.13 (–0.63, 0.37)	
	CES-D	20.35 (10.00)		N/A		N/A		14.68 (9.76)		–0.26 (–0.76, 0.25)	
**Service team (n=198)**											
	Use days	26.06 (5.25)		15.97 (10.51)		0.73 (0.36, 1.06)		17.37 (10.44)		0.34 (–0.18, 0.86)	
	CUDIT	20.71 (5.76)		17.19 (6.00)		0.52 (0.17, 0.86)		14.63 (6.66)		0.69 (0.15, 1.20)	
	SDS	8.06 (3.26)		6.29 (3.34)		0.63 (0.27, 0.97)		5.38 (3.24)		0.48 (–0.06, 0.99)	
	GAD-7	7.92 (4.97)		N/A		N/A		5.20 (4.24)		0.57 (0.04, 1.09)	
	ASRS	10.84 (4.11)		N/A		N/A		8.96 (3.92)		0.31 (–0.22, 0.82)	
	CES-D	23.51 (11.06)		N/A		N/A		16.10 (10.23)		0.26 (–0.27, 0.77)	

^a^Effect size Cohen *d* based on differences between the intervention and control groups.

^b^Previous 30 days according to the timeline follow-back method.

^c^N/A: not applicable.

^d^CUDIT: Cannabis Use Disorder Identification Test.

^e^SDS: Severity of Dependence Scale.

^f^GAD-7: Generalized Anxiety Disorder–7.

^g^ASRS: Adult Attention Deficit and Hyperactivity Disorder Self-Report Scale.

^h^CES-D: Centre for Epidemiological Studies Depression scale.

**Table 7:** Imputed data analysis.

Group and measure			Baseline			Posttreatment				Follow-up	
		mean (SD)		mean (SD)		d^a^ (95% CL)		mean (SD)		d (95% CL)	
**Internet as usual (n=189)**											
	Use days^b^	26.16 (5.53)		22.34 (9.47)		N/A^c^		21.98 (9.33)		N/A	
	CUDIT^d^	21.57 (5.30)		20.09 (6.31)		N/A		18.48 (6.31)		N/A	
	SDS^e^	8.12 (3.16)		7.67 (3.42)		N/A		7.38 (3.56)		N/A	
	GAD-7^f^	7.61 (4.54)		N/A		N/A		7.62 (3.84)		N/A	
	ASRS^g^	10.84 (4.21)		N/A		N/A		9.27 (4.04)		N/A	
	CES-D^h^	21.69 (10.12)		N/A		N/A		16.97 (9.66)		N/A	
**Social presence (n=188)**											
	Use days	24.92 (6.67)		16.88 (10.93)		0.48 (0.27, 0.68)		16.74 (10.54)		0.43 (0.22, 0.63)	
	CUDIT	20.79 (5.46)		17.07 (6.27)		0.50 (0.28, 0.69)		15.84 (6.68)		0.31 (0.10, 0.51)	
	SDS	7.38 (3.10)		5.58 (3.22)		0.46 (0.25, 0.66)		5.52 (3.34)		0.32 (0.12, 0.52)	
	GAD-7	7.42 (4.79)		N/A		N/A		5.48 (3.84)		0.42 (0.21, 0.62)	
	ASRS	10.69 (3.89)		N/A		N/A		9.82 (3.92)		–0.18 (–0.38, 0.03)	
	CES-D	20.35 (10.00)		N/A		N/A		16.74 (9.97)		–0.11 (–0.31, 0.11)	
**Service team (n=198)**											
	Use days	26.06 (5.25)		15.82 (10.59)		0.71 (0.50, 0.91)		16.21 (10.57)		0.60 (0.39, 0.80)	
	CUDIT	20.71 (5.76)		17.08 (6.46)		0.47 (0.26, 0.66)		14.56 (7.18)		0.49 (0.28, 0.68)	
	SDS	8.06 (3.26)		5.76 (3.32)		0.62 (0.40, 0.81)		5.16 (3.32)		0.60 (0.38, 0.79)	
	GAD-7	7.92 (4.97)		N/A		N/A		4.87 (3.90)		0.67 (0.46, 0.87)	
	ASRS	10.84 (4.11)		N/A		N/A		8.75 (3.82)		0.13 (–0.08, 0.32)	
	CES-D	23.51 (11.06)		N/A		N/A		16.04 (10.07)		0.27 (0.06, 0.43)	

^a^Effect size Cohen *d* based on differences between the intervention and control groups.

^b^Previous 30 days according to the timeline follow-back method.

^c^N/A: not applicable.

^d^CUDIT: Cannabis Use Disorder Identification Test.

^e^SDS: Severity of Dependence Scale.

^f^GAD-7: Generalized Anxiety Disorder–7.

^g^ASRS: Adult Attention Deficit and Hyperactivity Disorder Self-Report Scale.

^h^CES-D: Centre for Epidemiological Studies Depression scale.

### Adherence and User Satisfaction

Participants in the social presence group completed an average of 2.6 (SD 2.6) modules versus 2.4 modules (SD 2.5) completed in the service team group (t_374_=0.85, *P*=.20). Social presence group members (mean 46.9 minutes) spent significantly more time than service team users (37.5 minutes) on the program (t_374_=2.04, *P*=.02). Social presence group members exhibited the highest retention rate (29.8% versus 23.4% in the service team group), but this difference was not significant (χ^2^=1.81 *P*=.18).

There was no significant difference between the 2 intervention groups in level of user satisfaction (*P*=.83), but there were significant differences between each of the 2 intervention groups and internet as usual (internet as usual: mean 12.9, SD 6.9; social presence: mean 24.6, SD 5.4, t_139_=11.36, *P<*.001; service team: mean 25.5, SD 4.6, t_133_=12.74, *P<*.001).

Participants in the social presence group (mean 52.7, SD 13.6) scored significantly lower (t_114_=–2.81, *P*=.005) on the Working Alliance Inventory than those in the service team group (mean 59.6, SD 13.0).

### Adverse Effects

Among the 123 participants who completed the final follow-up assessment, 82 completed the questionnaire on adverse intervention effects (social presence: n=44, service team: n=24, internet as usual: n=14). Of these, 66 (80.5%) reported no negative effects during the study, while 10 (12.2%) answered that an adverse effect had affected them “somewhat negatively,” 4 (4.9%) answered that an adverse effect had affected them “quite negatively,” and 2 (2.4%) answered that an adverse effect had affected them “to a great extent.” However, there was no significant difference between the 3 treatment arms (χ^2^=1.33, *P*=.27).

### Dropout Analysis

Participants who dropped out scored significantly higher on the CES-D scale (t_197_=–2.21, *P*=.03), GAD-7 (t_218_=–2.92, *P*=.004), and ASRS (t_216_=–2.12, *P*=.04) scales; reported a greater number of risky alcohol use years (t_211_=–2.37, *P*=.02); finished fewer modules (t_140_=9.23, *P<*.001); and spent less time on the program (t_145_=7.15, *P<*.001) than those who completed the final follow-up evaluation. Full dropout analysis is summarized in Multimedia Appendix 2.

## Discussion

### Principal Findings

In this study, participants in intervention group service team (*d*=.60; *P*=.01) reported significantly greater reductions in cannabis use than those in the internet as usual group immediately after treatment and 3 months postbaseline. A reduction in the social presence group that was significant immediately after treatment (*d*=.48; *P*=.004) was no longer significant (*d*=.40, *P*=.07) at follow-up. Additionally, there were reductions in cannabis-use days 3 months after baseline in all 3 groups. There was no significant difference between the 2 active interventions.

The intervention group service team clearly outperformed all the internet-based interventions previously studied (Cohen *d* between 0 and 0.37 at follow-up 3 months from the start of treatment [19]), in terms of reducing cannabis use in general population samples, and the effects achieved were maintained 3 months after baseline. This persistence of effects aligns with the results of 2 previously published studies: one, an evaluation of video-based self-help [16], and the other, our own previous CANreduce 1.0 [14] study, in which we compared the efficacy of internet-based self-help with and without professional chat sessions to a control group. Within-group effect sizes for reducing cannabis use frequency (social presence: *d*_w_=.93; service team *d*_w_=.1.18) were better than those found in our previous study (*d*_w_=.75) [14] and similar to those found by Rooke et al (*d*_w_=1.08) [16]. Interestingly, the intervention group in the study [16] involved extended videos with an eCoach that, in our opinion, represents, apart from the reciprocal eCoach-participant relationship, the most important component of adherence-focused guidance. Thus, it appears that adherence-focused guidance also makes a difference during internet-based self-help, in terms of reducing cannabis use in cannabis users, which may allow findings from studies on stress and depression symptom reduction [25,26] to be expanded to the web-based cannabis interventions.

Our new programs also performed better than web-based interventions for brief personalized feedback (*d*_w_=.85) and extended personalized feedback (*d*_w_=.89) [48]; however, the study did not use a control group. Two recent meta-analyses [49,50] on brief interventions for cannabis reduction found little to no evidence of significant reductions in use or frequency. Even though some in-person brief interventions yielded small effects, the evidence consistently favored more intense, longer interventions [51]. There is evidence that brief interventions are beneficial for mild to moderate cases [52], while our program generated good effects in more severe cases. Current literature seems to indicate that more rather than less comprehensive interventions fare better in treating cannabis-use disorders. A combined stepped-care model with a range of varied intense treatment options could reach more users than a one-size-fits-all approach, as the majority of users are not in treatment [10].

Our a priori hypothesis that the social presence of a personal eCoach would outperform an impersonal study team was not confirmed. To the contrary, participants in the impersonal study team group had a significantly higher working alliance score with significant cannabis-use days reduction at 3 months (after baseline), indicating a stronger bond with the impersonal study team. We suspect that this is because the 2 adherence-focused guidance enhancement versions differed only slightly—the only differences being the presence versus absence of introduction videos and eCoach picture—and because support-team group participants may have perceived that there was an entire team being there to help them. Nevertheless, participants with access to an eCoach exhibited significantly greater adherence (in time spent, *P*=.02) and nonsignificantly greater retention (*P*=.18) than those with a support team, besides users in the latter group performing better overall in the primary outcome. Additionally, participants in the 2 intervention groups differed in their baseline scores for severity of dependence and depression. Thus, our findings did not support the existence of a linear relationship between adherence and treatment success. We found that greater adherence led to better retention and, with this, to greater data availability, and thus, more robust results to guide future research. A number of participants dropped out immediately after the start of the program, with 27% (102/386) not finishing a single module. This may stem from a discrepancy between what participants expected and what the program actually offered. Future programs should provide more information (eg, pictures or videos) to interested participants, so that they have a better idea of what to expect.

Our implementation of a single eCoach did not seem to achieve the level of social presence that was intended, even though it led to greater engagement. There may have been different intrapersonal aspects that could have affected how the participants perceived the eCoach or the study team. As we develop this program further, we intend to increase the program’s social presence and offer a variety of eCoaches to foster more personal freedom and choice. We nonetheless note that, in both intervention groups, the program was well received by users.

Among secondary outcomes, we found significant differences between the service team group and internet as usual were detected, in terms of reducing the severity of cannabis dependence (*P*=.02) and reducing cannabis-use disorder severity (*P*=.01). To our knowledge, this finding has only been reported once before, in a study [16] in which a similar program was evaluated.

We expected that the active interventions would significantly alleviate the symptoms of common comorbid mental health disorders more than internet access as usual was only partially correct, with greater reductions observed for general anxiety disorder.

In a meta-analysis [53], Kedzior and Laeber identified a positive association between both cannabis use and cannabis-use disorders, and anxiety disorders. Our findings that decreased cannabis use was accompanied by decreased anxiety symptoms were consistent with those of the meta-analysis [53]. It appears that cannabis has a bidirectional effect on anxiety [54]. On one hand, some individuals with anxiety may experience a degree of acute relief from their symptoms if they use cannabis infrequently and in low doses. On the other hand, regular and heavier use could lead to a cannabis-use disorder, thereby worsening anxiety symptoms. Interestingly, the acute effects of cannabis use, such as panic attacks, resemble the symptoms of anxiety disorders [55], which could have increased the anxiety score in our sample. This said, attributing anxiety to either an anxiety disorder or cannabis use does not make much of a difference to the person suffering from anxiety.

Measuring the success of the program in cannabis-use days may be not as specific as number of joints but corresponding results from CUDIT and the severity of cannabis dependence scale support the effectiveness of the program. Future usage quantity measures should also account for cannabidiol, as well as the potency of the tetrahydrocannabinol consumed. Measuring potency is difficult, as it requires either regulated products or toxicological testing, both of which seem unlikely to be feasible in Switzerland, Austria, or Germany for the foreseeable future. Future studies on adherence-focused guidance among cannabis users need to also start investigating long-term intervention effects (12 months or longer).

### Strengths and Limitations

CANreduce 2.0 was associated with comparable rates of adherence and retention but greater effect sizes than either the chat-enhanced or self-help only versions of CANreduce 1.0 [13], while being fully automated and requiring little to no human support, which in turn decreased the intervention’s complexity and costs of widespread implementation. Additionally, the program reached almost twice as many women (29.4% vs 17.4%), an older population (mean age 28.3, SD 7.9 vs 21.6 years, SD 8.4), and more severe users (daily use: 74.7% vs 31.2%) than Swiss outpatient treatment monitoring statistics [56]. These differences also were observed with the previous CANreduce 1.0 program [13], supporting our belief that such programs reach a different population of cannabis users than that reached by traditional outpatient treatment facilities.

This study has several limitations. First, technical difficulties decreased the number of participants who could be successfully followed up in the internet as usual group, which likely contributed to the overall high attrition rate (452/575, 70%), though this rate is common in these types of intervention [14,16]. To reduce any bias created by dropouts, multiple imputations were used, but large variances that decreased the chance of finding smaller effects still existed. Second, the introduction and rising popularity of low tetrahydrocannabinol (less than 1%) and high cannabidiol joints in the market may have caused some confusion in our study, as it is not clear how these were counted by participants; consequently, we had to drop our personalized standard joint measurement, in which participants chose from a range of different predefined joints to match their own consumed joints [32]. Third, all measures were self-reported and could not be validated externally, though there is evidence that the internet enables people to be more open and honest and to offer more accurate self-evaluations regarding their problems [57]. Last, the study only followed patients for a relatively short time period (3 months) meaning that we can make no claims of long-term treatment success; this said, beneficial effects were maintained for at least 3 months after the baseline was completed.

### Conclusions

The internet-based self-help interventions with an impersonal service team based upon adherence-focused guidance enhancement reduced cannabis use, severity of dependence, and general anxiety symptoms. The program reached a different group of treatment seekers in the general population than that reached by typical outpatient treatment options. The program is fully automated and requires little human support, which may render such programs cost-effective additions to the general health care system.

## Appendix

Multimedia Appendix 1 Introduction video of eCoach Lena.

Multimedia Appendix 2 Complete dropout analysis.

Multimedia Appendix 3 CONSORT-eHEALTH checklist (V1.6.1).

## Abbreviations

ADHD: attention deficit hyperactivity disorder
ASRS: attention deficit and hyperactivity symptoms self-report scale
CES-D: Centre of Epidemiologic Studies of Depression
CUDIT-R: Cannabis Use Disorders Identification Test Revised
DSM-IV: Diagnostic and Statistical Manual of Mental Disorders, 4th edition
GAD-7: Generalized Anxiety Disorder 7
ITT: intention-to-treat
MI: motivational interviewing
PTSD: posttraumatic stress disorder
SDS: Severity of Dependence Scale
