# Supplementary figures and images for: CANreduce 2.0 Adherence-Focused Guidance for Internet Self-Help Among Cannabis Users: Three-Arm Randomized Controlled Trial
Source: J Med Internet Res. 2021 Apr 30;23(4):e27463. doi: 10.2196/27463 (PMC8122293; doi:10.2196/27463)

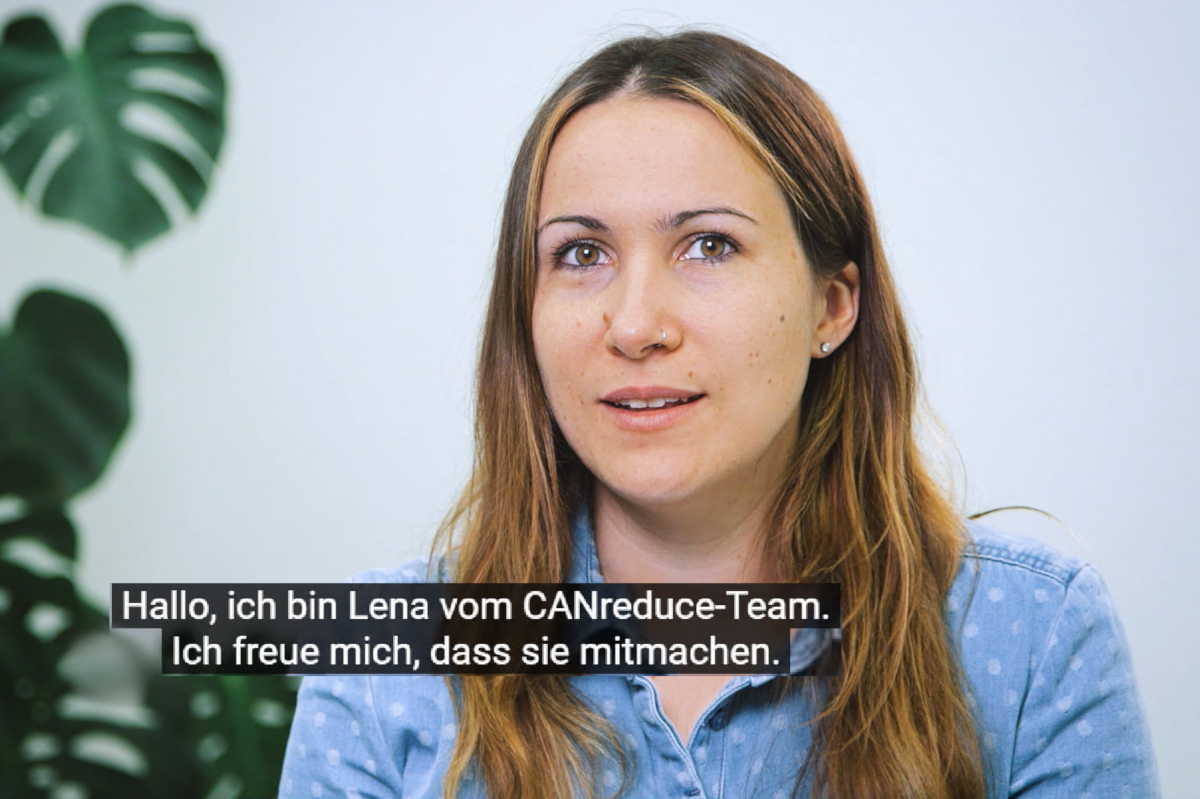

Supplement: Multimedia Appendix 1 [file jmir_v23i4e27463_app1.png]
