# Supplementary material for: CANreduce 2.0 Adherence-Focused Guidance for Internet Self-Help Among Cannabis Users: Three-Arm Randomized Controlled Trial
Source: J Med Internet Res. 2021 Apr 30;23(4):e27463. doi: 10.2196/27463 (PMC8122293; doi:10.2196/27463)
Supplement: Multimedia Appendix 2 [file jmir_v23i4e27463_app2.docx]

|  | | | | **Followed-up**  n = 123 | | | | **Dropped out**  n = 452 | | **Statistical Analysis** *(Chi-Square or t*-test*)* |
| --- | --- | --- | --- | --- | --- | --- | --- | --- | --- | --- |
| **Gender**, n (%) | | | |  | | | |  | | *Χ^2^(1, N=575) = 0.16, P = .156* |
| Female | | | | 43 (35.0) | | | | 126 (27.8) | |  |
| **Age**, *M (SD)* | | | | 28.3 (8.1) | | | | 28.2 (7.8) | | *t_187_ = 0.07, P = .944* |
| **Highest education**, n (%) | | | |  | | | |  | | *Χ^2^(5, N=575) = 6.93, P = .226* |
| **Country of origin**, n (%) | | | |  | | | |  | | *Χ^2^(3, N=575) = 4.64, P = .200* |
| **CES-D**, M(SD) | | | | 20.1 (10.3) | | | | 22.4 (10.5) | | *t_197_ = -2.21, P = .028** |
| **GAD-7** , M(SD) | | | | 6.6 (4.8) | | | | 7.9 (5.0) | | *t_218_ = -2.92, P = .004*** |
| **CUDIT**, M(SD) | | | | 20.5 (5.2) | | | | 21.1 (5.6) | | *t_205_ = -1.11, P = .267* |
| **SDS**, M(SD) | | | | 7.8 (3.4) | | | | 7.9 (3.1) | | *t_184_ = -0.235, P = .815* |
| **ASRS**, M(SD) | | | | 10.2 (3.6) | | | | 11.0 (4.2) | | *t_216_ = -2.12, P = .035** |
| **PTSD-7**, M(SD) | | | | 12.1 (4.9) | | | | 13.5 (5.1) | | *t_63_ = -1.59, P = .118* |
| **Number of Cannabis Joints**, M(SD) | | | | 21.3 (12.3) | | | | 22.8 (17.3) | | *t_267_ = -1.13, P = .256* |
| **Number of Consumption Days**, M(SD) | | | |  | | | |  | |  |
|  | Cannabis | | 25.4 (5.6) | | | | | | 25.8 (5.9) | *t_202_ = -0.78, P = .434* |
|  | Alcohol | | 8.2 (9.0) | | | | | | 7.5 (8.5) | *t_172_ = -0.79, P = .429* |
|  | Alcohol risky use^1^ | | 1.9 (3.8) | | | | | | 2.2 (4.6) | *t_200_ = -0.71, P = .480* |
| **Number of Consumption Years**, M(SD) | | | |  | | | |  | |  |
|  | | Cannabis | | | | 9.1 (7.6) | 8.2 (6.6) | | | *t_176_ = 1.17, P = .245* |
|  | | Alcohol | | | | 5.7 (7.6) | 5.0 (6.5) | | | *t_151_ = 0.89, P = .376* |
|  | Alcohol risky use^1^ | | | | 1.1 (3.9) | | | | 1.9 (3.7) | *t_211_ = -2.37, P = .019** |
| **Adherence intervention groups**, M(SD) | | | | | n = 102 | | | | n = 284 |  |
|  | Finished modules | | | | 4.6 (2.8) | | | | 1.8 (2.0) | *t_140_ = 9.23, P < .001**** |
|  | Time spent (minutes) | | | | 70.6 (49.8) | | | | 31.5 (38.1) | *t_145_ = 7.15, P < .001**** |

CES-D = Centre of Epidemiologic Studies of Depression Scale ; GAD-7 = Generalized Anxiety Disorder Screener Version 7; CUDIT = Cannabis Use Disorders Identification Test; SDS = Severity of Dependence Scale; ASRS = ADHD Self-Report Scale; PTSD-7 = Short Screening Scale for PTSD; ^1^Risky Use is defined as 5 or more standard drinks per day on at least 3 days a week. A standard drink is defined as 5 cl spirits, 15-20 cl wine or 33-45 cl beer
